# Sulphurous Acid as a Disinfectant

**Published:** 1882-07

**Authors:** 


### SULPHUROUS ACID AS A DISINFECTANT.

The *Mittheilungen aus dem Kaiserlichen Gesundheitsamte*, Bd. I., 1881, contains a paper by Dr. Wolffhügel, a distinguished pupil of Pettenkofer's, as to the value of sulphurous acid as a disinfectant. Recent discoveries in microzoic pathology have thrown such doubt on the efficacy of so-called disinfectants that the implicit confidence of the past seems likely to be succeeded by hopeless scepticism. The belief in sulphurous acid, however, is so deeply rooted in the professional as well as the popular mind, that it seems almost a pity to disturb it. Dr. Wolffhügel has undertaken, by a course of experiments, to ascertain the limits of its action, and the conditions most favorable thereto. For its production, he is content with the old method of burning stick sulphur, using a little spirits to set the combustion going, and fixes twenty grammes to each cubic metre of room space as the quantity necessary. In favor of the evaporation of sulphurous acid previously condensed to the liquid state are the absence of all risks of fire, and the greater amount that can be evolved in a given space, but in practice the cost would be a serious objection. Its activity is far greater in the presence of water, so that where bleaching or other destructive action is not feared, all articles exposed to it should be damped. Woollen fabrics absorb it far more than cotton or linen, but it does not penetrate to a sufficient depth into bales of goods or bundles of clothes, which should therefore be opened and spread out. In the dry state these are not appreciably injured by it. To aid its action, and to prevent its escape by diffusion, the walls and ceilings of rooms should be previously saturated with water Dr. Wolffhügel finds that in the utmost dilution (0.75 to 100 volume per cent) it speedily kills all bacteria and micrococci—as the bacillus of splenic fever, the cocci in putrid blood, etc.—in two minutes if wet, in twenty if dry; but that even in the highest state of concentration, as gas or in solution, it exerts no action whatever on spores. This is a most serious drawback, and he thinks that it would be well if we could abandon the use of all disinfectants that do not destroy spores as well as more developed organisms.—*Medical Times and Gazette*, April 20, 1882.

